# Anesthesia management in neonatal congenital bronchobiliary fistula: case report and literature review

**DOI:** 10.1186/s12871-020-01052-4

**Published:** 2020-06-02

**Authors:** Hong Yin, Guangyi Zhao, Yingjie Du, Ping Zhao

**Affiliations:** Department of Anesthesiology, Shengjing Hospital of China Medical University, Shenyang, 110004 China

**Keywords:** Congenital bronchobiliary fistula, CBBF, Neonate, Anesthesia, Chemical pneumonia, Hypoxemia, Hypercapnia

## Abstract

**Background:**

There is very little published literature and none that discussed care in a neonate regarding anesthetic risk and management of neonate with congenital bronchobiliary fistula during thoracoscopy and thoracotomy. This article analyzes related risk factors and literature review from perioperative ventilation, circulation and other aspects of management.

**Case presentation:**

A neonate diagnosed as congenital bronchobiliary fistula combined with severe chemical pneumonia, consolidation of the lungs, and infection was facing the risk of anaesthesia under thoracoscopy exploration surgery, who experiened more than 20 days diagnostic period before operation. Many risk factors have led to conversion from minimally invasive surgery to thoracotomy, including persistent hypoxemia, hypercapnia, difficult surgical exposure and extremly difficulty of intraoperative ventilation management. Anesthesia maintenance after conversion to open access remained problematic. Fortunately the patient showed no sign of any adverse CNS effects after 4 months of follow-up.

**Conclusions:**

The most prominent anesthesia challenges are hypoxemia, increased airway resistance, impaired ventilation, and the risk of metabolic acidosis. Close cooperation among the entire neonatal medical team is the key factors in successful management of this rare case.

## Background

Congenital bronchialbiliary fistula (CBBF) is a rare anomaly with low morbidity and very high mortality. CBBF is characterized by an abnormal communication between the respiratory tract (trachea or bronchi) and the biliary tract [1]. According to literatures, Only 45 cases of CBBF have been reported [2]. Surgical treatment is the only way to complete recovery. Because of the rarity of CBBF, there is very little published on anesthesia management other than a single case report but that this was in a 3 years old rather than neonate [3].

## Case presentation

**Fig. 1:**
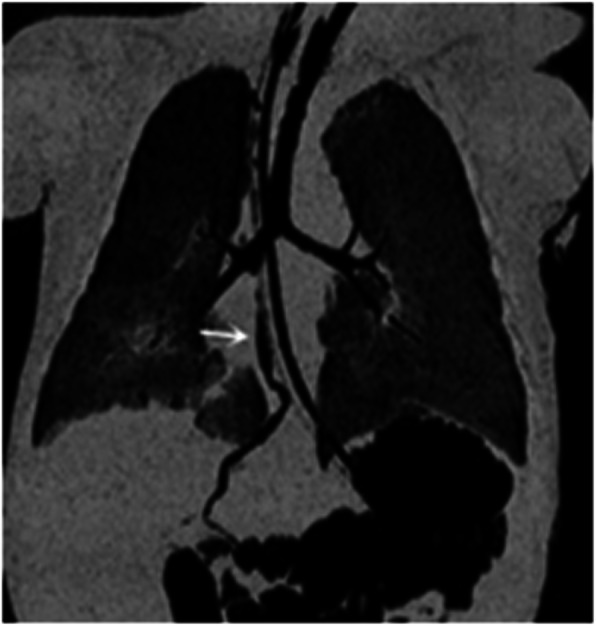
Three-dimensional chest computed tomogram showing the fistula originating from the right bronchus, travelling along the esophagus, and passing through the diaphragm and into intrahepatic biliary tract at the site of the esophageal hiatus

**Fig. 2:**
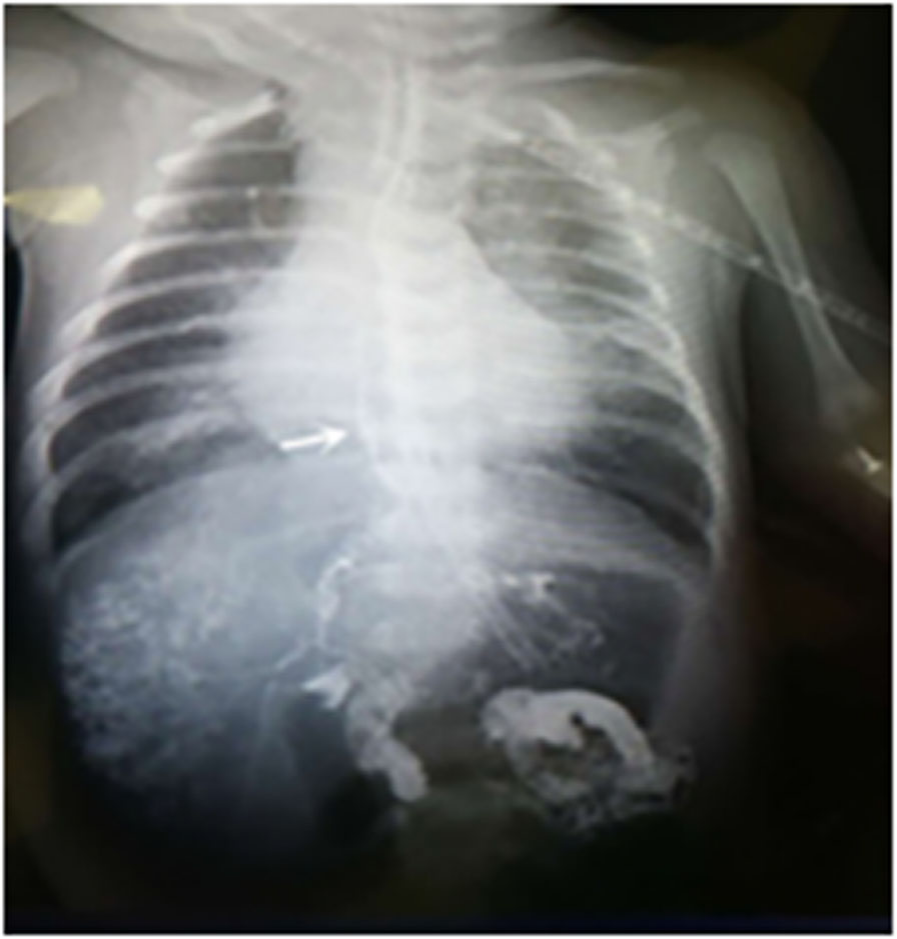
Bedside x-ray showing contrast flowing from the abnormal opening in the right main bronchus through the fistula and into the biliary tract, the gall bladder, and the duodenum. Contrast study via fiberoptic bronchoscope: contrast is seen in the fistula, the biliary tract, and the duodenum. The white arrow shows the fistula

A 5-day old full-term female baby, delivered by cesarean section, 2880 g of birth weight, was referred to our hospital because of intermittent cyanosis. She had frequent choking with an excessive volume of yellowish-green saliva at the third day of born. Airway examination showed normal mouth opening and neck movements with no facial anomalies. After admission, her respiratory function continued to deteriorate and endotracheal intubation and ventilator assistance became necessary. Tracheal secretion of a large volume of yellowish-green fluid persisted and three-dimensional computed tomography (3D-CT) of the chest showed a fistula originating from the right bronchus, running distally along the esophagus, and passing through the diaphragm and into the intrahepatic biliary tract at the site of the esophageal hiatus. There was air in the intrahepatic biliary tract and in the common hepatic duct, along with a bilateral, mainly lower-lobe, pneumonia (Fig. 1). CBBF was diagnosed accordingly. Ultrasonography and bronchoscopy confirmed the CT findings. The flexible fiberoptic bronchoscopy identified the opening of the fistula in the right main bronchus. A contrast x-ray examination of the fistula was performed during bronchoscopy. Contrast flowed from the opening in the bronchus through the fistula and into the biliary tract, the gall bladder, and the duodenum (Fig. 2). So the preoperative diagnosis was: 1. bronchobiliary fistula; 2. neonatal pneumonia; 3. gastroduodenal reflux; and 4. congential heart disease.

Preoperative treatment measures included CPAP ventilation (see Table 1 for blood gas results), gastrointestinal decompression and fasting, inhibition of gastric acid secretion, promotion of gastric peristalsis, antibiotics, parenteral nutrition, myocardial nutrition, sedation analgesia, and precise fluid management and keep elevation position to encourage consolidation of the biliary contents to distal lung. Due to the rarity of the disease, it was spent in the preoperative diagnosis process for a relatively long time after hospitalization. And the baby was scheduled for endoscopic cholangiography and thoracoscopic right bronchobiliary fistulectomy under general anesthesia on the second day after the diagnosis.

**Table 1:** Arterial blood gas analysis at different time points

Time Point	PH	PCO2	PO2	HCT	tHb	GLU	LAC	HCO3-	BE
		(mmHg)	(mmHg)	(%)	(g/dl)	(mmol/l)	(mmol/l)	(mmol/l)	(mmol/l)
Preoperation	7.37	46.3	55.9	33.1	10.6	5.2	1.5	26.3	2.9
During thoracoscopy	7.37	41.1	45.8	37.3	11.8	4.6	1.1	2.3	−2.26
During open surgery	7.21	39.3	55.6	41.7	13.6	8.2	4.5	15.3	−10.4
End of operation	7.36	32.8	97.6	34.3	11.4	5.8	2.0	19.3	−6.27
Entering NICU	7.38	45.7	64.2	43.9	15.0	5.6	1.5	21.1	−2.15

### Anesthesia

**Fig. 3:**
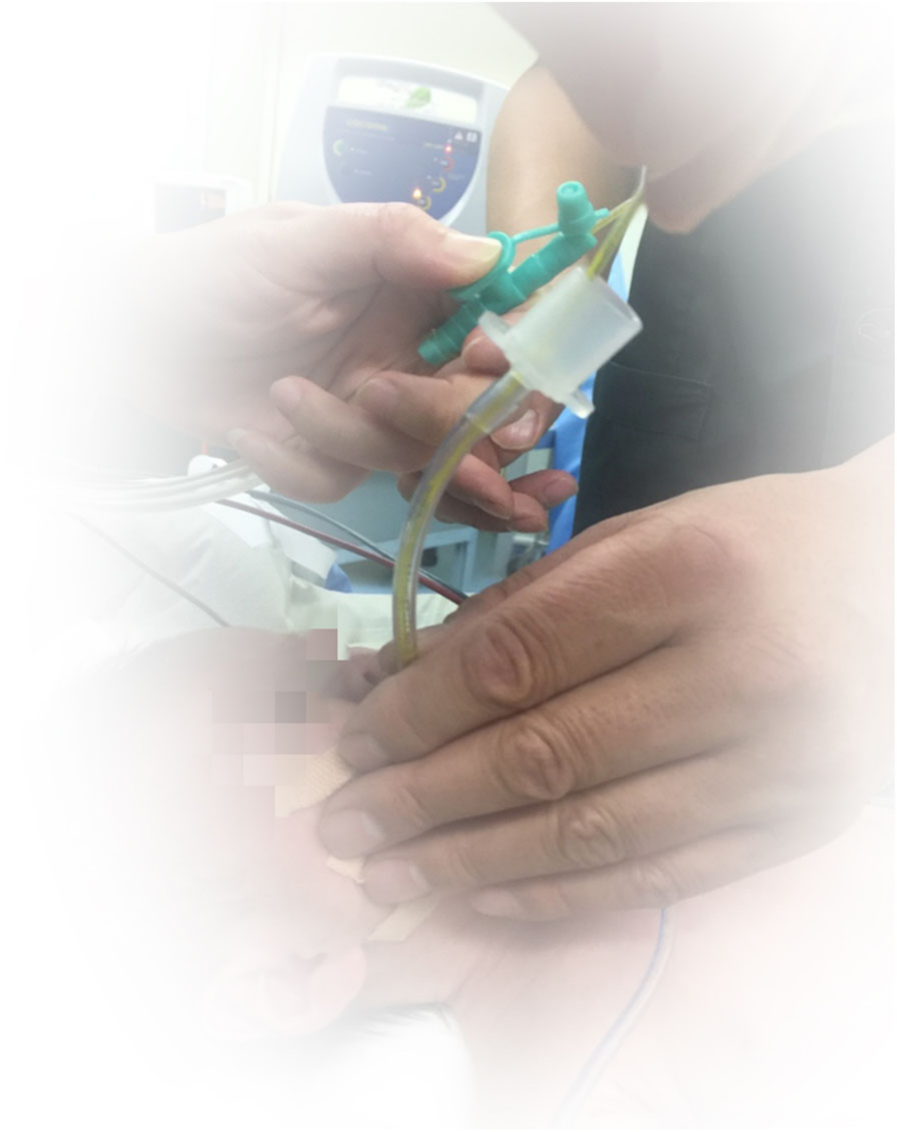
Large amounts of bilious aspirate were cleared via endotracheal suction

**Fig. 4:**
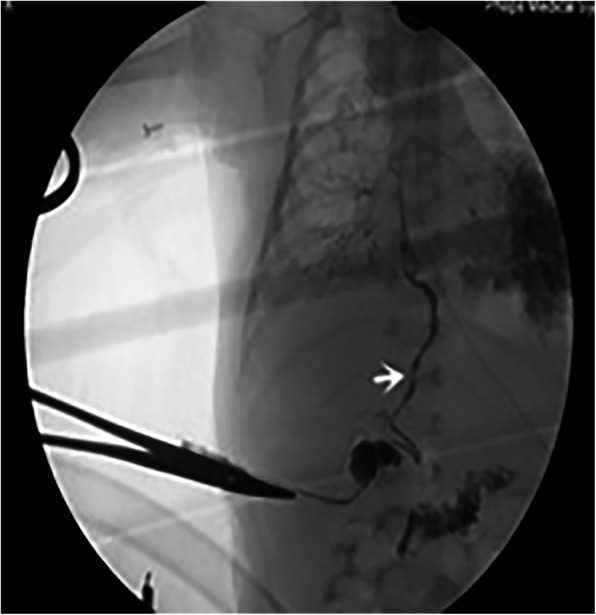
Intraoperative cholangiography shows the connection between the biliary tract and the right main bronchus. The distal bile duct is patent. The white arrow indicates the biliary tract

Anesthesia: The patient was 16 days old on the day of surgery with 2.2 kg of body weight, and golden-yellow lung secretions persisted (Fig. 3). Taking into account the preoperative examination confirmed that the fistula is away from the tracheal carina, and the fact that CPAP had no obvious abdominal distension symptoms before surgery, the tracheal intubation catheter is placed in the main airway, away from the fistula. In view of the tracheal intubation when entering the operating room, we take more safer method of inhalation anesthesia induction. During anesthetic maintenance, sevoflurane combined with intravenous remifentanil was adopted. In addition to regular monitoring, invasive arterial pressure monitoring, intermittent blood gas analysis, and continuous cerebral oxygen saturation (ScO_2_%) monitoring were also performed. Due to the lack of neonatal bronchial obstruction at the time, we did not perform lung isolation techniques and respiratory management as planned [4, 5]. The lungs, particularly the left lung, were infiltrated with iodinated contrast agent during contrast examination of the biliary tree (5 mL intrahepatic iodine tracer), and this was mostly cleared by immediate endotracheal suction. The contrast examination confirmed the right-sided bronchobiliary fistula and showed a normal extrahepatic biliary tract (Fig. 4). Bilateral lung sounds returned to baseline after suctioning of the contrast agent from the lungs and the SpO_2_ remained above 85%. At this point, thoracoscopic fistula resection was attempted. The initial pneumothorax parameters were: CO_2_ inflation pressure 3 mmHg with flow rate adjusted to 1 to 2 L/min under pressure controlled ventilation with PIP 20 to 23, PEEP 4 to 5 mmHg, frequency 30 to 32 breaths/min, and I:E 1.5 to 1.8. In spite of the normal bronchial breath sounds after suctioning, there was persistent hypoxemia and increased airway resistance during thoracoscopy. Intermittent manual assist ventilation was adopted to maintain oxygenation to the greatest extent possible to ensure that cerebral oxygen saturation did not fall below 20% of baseline. But finally, considering the safety of the patient and difficult exposure the procedure was converted to an open access. Arterial blood gas analysis before, during, end operation, during thoracoscopy、thoracotomy and at the time of entering NICU are summarized in the Table 1.

Ventilator management during thoracotomy remained problematic, with high airway pressure, low tidal volume, and intermittent hypoxemia during thoracotomy. Breathing parameters include pressure-controlled ventilation combined with intermittent manual inflation management to maximize tidal volume and inhalation time to ensure oxygen supply. And the peak inspiratory pressure was limited to less than 28 mmHg. The patient developed a metabolic acidosis with base excess = − 11 and lactate = 4.5, which was corrected by intravenous sodium bicarbonate infusion. In view of the long operation time, about 8 ~ 10 ml/kg of intravenous isotonic saline including glucose (1 ~ 2% isotonic solution with sugar) supplementation and equal volume of RBC transfusions according to blood loss were given to maintain effective perfusion of the microcirculation and adequate blood pressure and urine output. Phenylephrine was prepared to maintain the blood pressure within 20% of the baseline value if needed. The ScO_2_% fluctuated in response to fluctuations in the SpO_2_, and at one point, the ScO_2_ fell to 38% (basal value 58%). Although this was a fairly short-term episode, it is nonetheless worthy of attention. When the SpO_2_ improved, the ScO_2_% returned to approximately 50%.

A retrograde methylene blue injection was performed for precise positioning of the fistula, and once again, both lungs were infiltrated and immediate suctioning was required. The total operative time was approximately 5.5 h, and the infant remained intubated postoperatively and was transferred to the neonatal intensive care unit (NICU) after surgery. Postoperative histopathological examination confirmed that the resected fistula originated from the bile duct. Biliary epithelial cells were found at the margin of the bronchial fistula and there was interstitial cholestasis.

### Postoperative course

Normal oxygenation and arterial blood gases were maintained by CPAP ventilation with PEEP of 5 cm H_2_O and FiO_2_ of 0.4 (see Table 1 for blood gas values). Prolonged postoperative ventilator support was necessary because of persistent pneumonia, and the initial attempt to remove the thoracic tube failed because of acute pneumothorax and subcutaneous emphysema. The patient also had cholestasis and developed a chylothorax during the recovery stage. Drainage of chylous fluid from the thoracostomy tube gradually diminished as gastrointestinal decompression and use of intravenous methylprednisolone, NPO, and parenteral nutrition with antibiotic therapy were continued, and oral feeding was resumed without adverse reactions once the thoracic drainage was minimized. After 43 days in hospital, the infant recovered well and was stable for discharge. There were no neurological complications during follow-up after 4 months.

## Discussion and conclusion

Congenital bronchobiliary fistula (CBBF) is a rare malformation with low morbidity and high mortality. When CBBF is suspected, the diagnosis can be confirmed by MRI, 3D-CT, or bronchoscopy, and ultrasound can also provide valuable clues. Bronchoscopy, which can show the bronchial opening of the fistula and the flow of bile through that opening, is the most common method of diagnosis [6–8]. Definitive treatment of CBBF requires surgical resection [1]. From the relatively long period of diagnosis of this case and clinician lacks diagnostic experience, it fully illustrates the rarity of the disease. Relatively long diagnosis time exacerbates lung inflammation and changes in lung consolidation.

Although there have now been dozens of repairs reported, so far only one, in a 3-year old girl (weight 10 kg), has focused on anesthesia management [3], and perioperative anesthesia management in a low birthweight neonate with impaired respiratory physiology would be different [9]. Low birth weight and incomplete lung development are only two of the many challenges in anesthesia management during resection of CBBF in neonates. CBBF presents with nonspecific symptoms, and recurrent chest infection, fever, dyspnea, and pulmonary consolidation may all be signs of the anomaly, which may also be accompanied by congenital heart defects. All of these factors will affect the anesthesiologist’s decision-making during the perioperative management of cardiovascular and respiratory function [10–13]. In our case, the patient’s already compromised lung function was further compromised after both lungs were infiltrated by the contrast agents that were necessary to locate and identify the fistula, and this caused further difficulty in providing effective ventilation and resulted in obstinate hypoxemia. Fortunately, there was some improvement after suctioning and manual-assist ventilation. In addition, surgery times may be prolonged during resection of CBBF, particularly when there are other congenital biliary defects to be repaired. Anesthesia management is also particularly challenging when surgery is performed in neonates less than 1 month after birth and in the presence of congenital heart disease and bilateral lung consolidations [14].

Given that the main problems during the perioperative period are inadequate ventilation and hypoxia, 116 papers can be retrieved according to search strategy ((((Anesthe*[Title] AND (Chemical pneumonia OR Hypoxemia OR Hypercapnia OR dyspnea))) OR (Anesthe*[Title] AND thoracoscop*)) OR (bronchobiliary fistula AND Anesthe*[Title]) Filters: English; Infant: birth-23 months in on pubmed database. After excluding literatures which are not related to newborns and infants and or not related to perioperative anesthesia management, 25 related literatures are finally obtained (Table 2). But there is only one article describing directly the anesthesia of neonatal tracheobiliary fistula, which is a short report of a three-year-old child [3].

**Table 2:** Literature search results related to anesthesitic management of tracheobiliary fistula operation in children

Year	Author	Research type	Operation type	Age	Weight	Number of patients	focus point
2018	Mohtar S [4]	Retrospectively	Thoracoscopy	< 3 months	1–21 kg	60	Extraluminal bronchial blocker
2018	Vika Fatafehi [9]	Retrospectively	Surgery lasting < 45 min	Pediatric	–	1090	Desaturation
2019	McCann ME [10]	Review	All	Infant	–	–	Brain injury, CBF
1998	Semin Perinatol [15]	Review	All	Micropremie	–	–	Anesthetic considerations
2016	King MR [11]	Retrospective analysis	All	1–20 Y	1–120 kg	446	Desaturation
2015	Wei K [16]	Case report	Emergent inguinal herniorrhaphy	21 Ds	3.8 kg	1	Hypercapnia
2011	Broemling N [17]	Review	Thoracic/thoracotomy	Neonate	–	–	anesthetic management
2005	Choudhry DK [5]	Review	Thoracic surgery	Infants and Children	–	–	Single lung ventilation devices
2005	Krosnar S [18]	Retrospective	Thoracoscopic surgery	Children	–	8	Anesthetic management of TEF/EA
2016	Razlevice I[]26	Prospective study	Thoracic or urologic surgery	< 3 months	–	44	Cerebral oxygen desaturation
1972	Smith AL [19]	Review	All	–	–	–	Cerebral blood flow and metabolism
2008	Sola A [20]	Review	All	Neonate	–	–	Oxygen toxicity
1994	Keenan RL [21]	Retrospective analysis	Noncardiac surgery	0–1 Y	–	4645	Bradycardia during anesthesia in Infants
2004	Means LJ [22]	Review	Laparoscopic/thoracoscopic surgery	–	–	–	Anesthetic management
2010	De Armendi AJ [23]	Case report	Proximal hypospadias repair	8 M	8.4 kg	1	Hypercapnia
2015	Ehlers M [12]	Case series	Thoracic surgery	0–3 M	2.1–4.0 kg	3	Hypercapnea and low cardiac output
1999	Petrat G [24]	Review	Minimally-invasive surgery	–	–	–	Anesthetic considerations
2016	Chiluveru SA [16]	Case report	Right lobectomy for CPAM	6 M	4 kg	1	Pulmonary hypertension
1993	Tobias JD [25]		Laparoscopic and Thoracoscopic procedures	Children	–	–	Air embolism
1999	Gunawardana RH [26]	Prospective study	Cleft lip and palate surgery	3 M-5Y	–	200	Low HB and brydicardia
1999	Chen KB [27]	Case report	Thoracotomy	Preterm infant	500 g	1	Preterm infant anesthesia of PDA
2008	McHoney M [13]	Prospective study	Thoracoscopy	1D-15Y	–	–	CO2 excretion, thermoregulation
1992	Kinouchi K [14]	Prospective study	All	1 M-12Y	–	61	Respiratory infection
1980	Welle P [28]	Prospective study	No abstract	Neonate	–	–	Transcutaneous oxygen monitoring
1994	Rowe R [29]	Case series report	Thoracoscopy	Pediatric	–	9	Anesthetic management

*M* months; *D* days *Y* years; *CPAM* congenital pulmonary airway malformation; *CBF* cerebral blood flow; *TEF/EA* tracheoesophageal fistula; *HB* hemoglobin; *PDA* patent ductus arteriosus

The reasons for intractable hypoxemia during surgery in the present case, which presented as intraoperative hypoxia, metabolic acidosis, and finally an inability to tolerate thoracoscopic surgery, requiring conversion to open thoracotomy, included the following:

1. Although auscultation of the lungs indicated normal air movement with only slightly coarsened breath sounds during thoracoscopy, elevated airway pressures and low tidal volume with hypercapnia were noted. Ventilation was acceptable after conversion to thoracotomy, but the bilateral chemical pneumonitis combined with acid reflux pneumonia and partial lung consolidations, which are characterized by inflammatory cell infiltration, alveolar epithelial cell destruction, degeneration, increased capillary wall permeability, interstitial pulmonary edema, and thickened, edematous alveolar membranes, ultimately caused insufficient ventilation leading to hypoxemia that severely compromised the PaO_2_ [30].
2. The establishment of an iatrogenic pneumothorax followed by surgical compression of the lungs during thoracotomy led to atelectasis, reduced respiratory area, and hypoxia. Compression of the mediastinum during thoracotomy caused the heart rate and blood pressure to plummet twice, but the heart rate and blood pressure recovered spontaneously as soon as the compression was relieved.
  Intraoperative problems that are commonly reported during thoracoscopic surgery include desaturation, transient hypotension requiring vascular expansion, hypercapnia (> 45 mmHg), hypothermia (< 34.9 °C), and metabolic acidosis [22]. Some of these require conversion to an open approach, while some are corrected without interruption of surgery. Significant risk factors for complications during thoracoscopic resection of CBBF also include young age of the patient, low body temperature, thoracic insufflation, high insufflation pressure and flow, and length of surgery [16, 18, 24, 27, 31]
3. The presence of a bronchobiliary fistula causes direct air leak from the fistula into the biliary system during mechanical ventilation, which further contributes to decreased effective ventilation and low PaO_2_.
4. V/Q mismatch occurs during pneumothorax or open thoracotomy in a lateral position because of reduced perfusion at the lung bases, and this can also contribute to insufficient ventilation in neonates, with a decrease in total tidal volume causing deficient minute ventilation and hypoxemia [32, 33].
5. Tracers that are used to locate the bronchial opening and trace the fistula may infiltrate the lungs, causing transient increase in airway pressures and reduced oxygenation. In our case, these adverse effects on respiratory gas exchange were partially ameliorated by immediate suctioning and by the use of the lowest effective doses contrast agent and methylene blue.
6. Congenital heart diseases, such as atrial septal defect, can cause pulmonary hypertension. Right-to-left shunt occurs during hypoxia and CO_2_ accumulation causes refractory hypoxemia. In this case, the reasons for the difficulty in keeping the SaO_2_ above 85% were complex, and whether there was a right-to-left shunt remains unknown. Perioperative echocardiography or TEE may assist in diagnosis, but these were not performed in this case [17, 34–36].

Finally, intraoperative monitoring of cerebral oxygen saturation provided strong evidence of perioperative balance of cerebral oxygen supply and demand. Noninvasive real-time monitoring is an advantage in the neonate [37]. A baseline cerebral perfusion value of 25%, or > 250 min of the area under the AUC curve, is thought to be of clinical value as a predictor of an increased risk of postoperative neurological complications. Cerebral oxygen saturation is related to Hb, NIBP, PaO_2_, PaCO_2_, and other factors. Elevated PaCO_2_ during cerebral hypoxia stimulates cerebral vasodilation and increases cerebral perfusion. However, cerebrovascular autoregulation is limited in neonates, and additional care must be taken during the perioperative period to monitor and maintain the balance of cerebral oxygen supply and demand in this especially vulnerable group [20, 38, 39]. Fortunately, in the present case, after 4 months of follow-up, the patient showed no sign of any adverse CNS effects.

In summary, for newborns with CBBF, a rare anomaly with a high mortality rate, for which surgery is the only definitive treatment, the most prominent anesthesia challenges are hypoxemia, increased airway resistance, impaired ventilation, and the risk of metabolic acidosis, particularly during prolonged surgeries. Careful perioperative anesthesia management and close cooperation among the entire neonatal medical team are the key factors in successful management of this rare condition. Traditional thoracotomy may be safer and more appropriate for neonate combined with extremely risk factors.

## Data Availability

Data are available on request due to privacy or other restrictions. The data that support the findings of this study are available on request from the corresponding author PZ. The data are not publicly available due to them containing information that could compromise research participant privacy/ consent.

## Abbreviations

CBBF: Congenital bronchialbiliary fistula
ScO2%: Cerebral tissue oxygen saturation
SpO2: PULSE oximetry saturation
PIP: Peak airway pressure
PEEP: Positive end expiratory lung pressure
CPAP: Consistent positive airway pressure
